# The effect of nano/microparticles of bee pollen on the shelf life of high‐fat cooked sausage during refrigerated storage

**DOI:** 10.1002/fsn3.4086

**Published:** 2024-03-07

**Authors:** Zahra Mashhadi, Nafiseh Davati, Aryou Emamifar, Mostafa Karami

**Affiliations:** ^1^ Department of Food Science and Technology, Faculty of Food Industry Bu‐Ali Sina University Hamedan Iran

**Keywords:** antimicrobial, antioxidant, bee pollen, sausage

## Abstract

Sausage is susceptible to oxidative changes in lipids and microbial spoilage due to the presence of water, fat, protein, and vitamins. Bee pollen (BP) as a source of potential antioxidants and antibacterial compounds can effectively prevent lipid peroxidation and microbial spoilage in meat products. The aim of the present study was to investigate the antibacterial and antioxidant activities of BP and the effects of nano/microparticles of bee pollen extract (n/m BP) at a concentration of 125 and 250 mg/100 g meat on the oxidative stability and microbial growth of high‐fat sausage during 30 days of storage at 4°C. The formation of BP particles in the nano/micro range was confirmed by scanning electron microscopy. High concentrations of total phenolic compounds (28.26 ± 0.10 mg GAE/g BP) with antioxidant activity (EC_50_ = 5.4 ± 0.07 mg/mL) were detected in BP. Based on the microdilution assay, the minimum inhibitory concentration of n/m BP for all test bacteria was 1000 (μg/mL) and the minimum bactericidal concentration of n/m BP was 2000 (μg/mL) for *Staphylococcus aureus* and *Bacillus cereus* and 4000 (μg/mL) for *Escherichia coli* and *Pseudomonas aeruginosa*. The n/m BP treatment (250 mg/100 g meat) showed a higher pH value (*p* < .05) and lower TBARS values (*p* < .05) than the ascorbic acid treatment (100 mg/100 g meat) and the control during the storage period. The microbial analysis showed that the addition of n/m BP led to a significant decrease (*p* < .05) in the total bacterial count, coliforms, *S. aureus*, and fungal population compared to the other samples. The results show that the addition of n/m BP (125 mg/100 g) can improve the texture, taste, and overall acceptability of the sausage compared to the control sample. In conclusion, this study suggests that BP can replace synthetic antioxidants in high‐fat sausages at the nano/microparticle level.

## Graphical abstract

There is an increasing tendency to use natural antioxidants such as bee pollen (BP) in meat products. BP at the nano/microparticle scale has strong antioxidant and antimicrobial activities. Nano/microparticles of BP can effectively prevent lipid oxidation and control the microbial growth of high‐fat sausage.

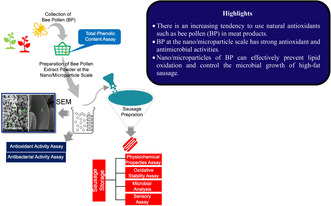

## 1 INTRODUCTION

Processed meat is now an important part of the diet in modern societies (Novaković et al., 2021). Sausages are known to be one of the most consumed meat products in the world (Fernández‐López et al., 2019). Fatty meat products, such as high‐fat sausages, always have their fans because of their special taste, which is due to the lipid compounds. Fat is an unavoidable component in the preparation of meat products and influences the sensory attributes, quality, and shelf life of the product (Wenjiao et al., 2014). Meat products with a high‐fat content are often rancid, which is caused by the oxidation of polyunsaturated fatty acids that are exposed to oxygen, heat, moisture, or an enzyme reaction during storage (Falowo et al., 2014; Georgantelis et al., 2007). This oxidation is recognizable by the formation of end products that result from advanced lipid oxidation and carry numerous odor and taste molecules (Wenjiao et al., 2014; Xiong, 2017). Malonaldehyde is known as an indicator of oxidative stress in the differentiation of lipid peroxidation in meat products (Fisch et al., 2003; Guyon et al., 2016). It should be noted that the membrane of muscle cells is damaged during the meat grinding process, which allows interaction between pro‐oxidants and unsaturated fatty acids (UFA) and accelerates lipid oxidation (Min & Ahn, 2005). All meat products contain antioxidants that inhibit lipid peroxidation. In general, synthetic antioxidants including propyl gallate, butylated hydroxytoleune (BHT), tert‐butylhydroquinone, and butylated hydroxyanisole are used to prevent the oxidation of fats and improve the sensory properties of processed foods (Mohdaly et al., 2010; Pateiro et al., 2018; Shah et al., 2014). However, both manufacturers and consumers are calling for synthetic antioxidants to be replaced by natural antioxidants. Due to the harmfulness of synthetic antioxidants and the growing request for healthy foods, there is an increasing tendency to consume natural antioxidants as a good alternative (HaÅ et al., 2011; Pateiro et al., 2018; Shahidi & Zhong, 2010). There are several studies demonstrating the effect of natural antioxidants from garlic (Horita et al., 2016), walnut green husk (Salejda et al., 2016), rosemary (Liu, Tsau, et al., 2009), decoctions of various mushrooms (Novakovic et al., 2020). In addition to oxidative spoilage of lipids, there is always a risk of food poisoning from the consumption of contaminated meat products, according to the reports of legal authorities (European Food Safety Authority and European Centre for Disease Prevention and Control, 2019). Therefore, processed meat producers are forced to use chemical preservatives such as sodium nitrite to control the growth of pathogenic bacteria such as *Clostridium botulinum* and *Listeria monocytogenes* (de Cássia Aleixo et al., 2022; Fernández et al., 2023). There are several studies showing the addition of natural additives to control the microbial growth of meat products. This applies to resveratrol and thymol essential oil in cooked sausage (Hashemi et al., 2023), dietary fiber in cooked sausage (Aminzare, Hashemi, Afshari, Noori, & Rezaeigolestani, 2022), edible chitosan coating with resveratrol and essential oil of *Satureja bachtiarica* in fresh chicken meat (Abdalbeygi et al., 2022), essential oil of *Ziziphora tenuior* and orange fiber in cooked beef sausage (Aminzare, Hashemi, Afshari, Mokhtari, & Noori, 2022) and nanoemulsion of essential oil of *Zataria multiflora* enriched with cinnamaldehyde in fresh chicken meat (Abbasi et al., 2021). Recently, studies have shown that bee pollen (BP) is effective on the shelf life of meat products such as sausage and black pudding (Anjos et al., 2019; Novaković et al., 2021). BP, known as bee bread and ambrosia, is a collection of flower pollen from different plants and packed by worker honeybees. This product is used as a primary nutrient substance for the inhabitants of the hives (Gilliam, 1979; Komosinska‐Vassev et al., 2015). BP is known as a functional ingredient due to the presence of nutrients compounds such as vitamins, proteins, lipophilic carotenoids, free sugars, carbohydrates, lipids, minerals, phenolic compounds (quercetin and its derivatives), sterols, terpenoids, and flavonoids (Bogdanov, 2012; Kostić et al., 2019; Margaoan et al., 2014). In addition, BP is the focus of researchers' attention due to its biological activities such as its antioxidant effect (Carpes et al., 2013; de Florio Almeida et al., 2017; Estevinho et al., 2019; Krystyjan et al., 2015), antimutagenic (Tohamy et al., 2014), anti‐inflammatory (Maruyama et al., 2010), and antimicrobial (Morais et al., 2011; Pascoal et al., 2014) properties. Several studies have been conducted to fortify foods such as bread, meat products, fruit juice, biscuits, and milk with BP (Anjos et al., 2019; Kostić et al., 2020). So far, meat products such as frankfurters (Novaković et al., 2021), refrigerated sausages (de Florio Almeida et al., 2017), black pudding (Anjos et al., 2019), and meatballs (Turhan et al., 2017) have been enriched with BP due to its antioxidant effect as a free radical scavenger that delays lipid oxidation during storage. It should be noted that the bioactive compounds at the nano/microparticle scale can be used more effectively to extend the shelf life of foods than larger particles due to the larger contact surface between the bioactive ingredients and the product matrix so that their antioxidant and antibacterial properties are enhanced (Jadhav et al., 2023; Otoni et al., 2014). However, as far as we know, the addition of BP extract at the nano/microparticle scale to full‐fat sausages has not yet been investigated. This study aimed to evaluate the shelf life of high‐fat sausage using nano/microparticle‐scale BP extract powder (n/m BP) as a natural antioxidant and antimicrobial agent. For this purpose, the antibacterial and antioxidant activities BP were determined. Subsequently, different formulations of high‐fat sausage with n/m BP and ascorbic acid as a synthetic antioxidant were prepared to evaluate the effects of n/m BP on the shelf life, physicochemical, and sensory properties of the high‐fat sausage during refrigerated storage.

## 2 MATERIALS AND METHODS

### 2.1 Materials

The BP was supplied from local beekeepers in Arak, the central region of Iran, in the Autom of 2022 and stored in a dry, dark, and cool place until further analysis. All chemical compounds, solvents, and reagents were prepared by Sigma‐Aldrich Co. from the United States and Merck Co. from Darmstadt, Germany.

### 2.2 Preparation of n/m BP

To prepare the BP extract, 40 g of BP was mixed with 400 mL of ethanol 80% (v/v) in a shaker (Daneshvar, Tehran, Iran) at 40°C for 1 h at 150 rpm in a dark place. The mixture was filtered through Whatman filter paper (No. 1) and then centrifuged at 2473 *g* for 10 min (UNIVERSAL 320 R, swinging bucket rotor 1351, Andreas Hettich GmbH, Germany). The upper aqueous phase was removed as an extract and the solvent was removed using a rotary evaporator (Ev311H, LabTech, Sorisole, Italy) at 70 rpm for 2 h (de Florio Almeida et al., 2017; Mohdaly et al., 2015). To prepare n/m BP, 75 g of the extract was made up to 2500 mL with 150 g of milk powder and distilled water. Then, the resulting solution was sonicated (400UPS1, Fapan, Tehran, Iran) (40 kHz, 100 W) for 20 min and finally injected through the nozzle into the mini spray dryer model B‐191 (UPWR‐TN‐555, ToosNano, Mashhad, Iran) with the following conditions: Inlet temperature of 130°C; feed rate of 100 mL/h; column temperature of 40°C; pressure of 1 bar. The dried BP was stored at −20°C until further analysis.

### 2.3 Scanning electron microscope (SEM) of BP particles

The morphology and size of the BP particles were analyzed using the SEM (FEI Model Quanta 450 FEG, Hillsboro, OR). The BP particles were sputtered with a thin layer of gold and the SEM images were recorded at a voltage of 25.0 kV and then viewed at 15,000–16,000× magnification.

### 2.4 Total phenolic content (TPC) and antioxidant activity of n/m BP

The total phenolic content of BP was determined using the Folin–Ciocalteu assay according to Singleton et al. (1999); Moreira et al. (2008) with modification. First, 0.5 g n/m BP was dissolved in 10 mL of 70% ethanol, then 1 mL of this solution was mixed with 2.5 mL of Folin–Ciocalteu and 2 mL of 4% sodium carbonate (v/v) and stored for 2 h in the dark. The absorbance of n/m BP was measured using a UV–VIS spectrophotometer (XD7500, LOVIBOND, Dortmund, Germany) at 760 nm. After preparing standard solutions of gallic acid, a calibration curve was established in the concentration range of 5–30 μg/mL (Zugazua‐Ganado et al., 2024). The results were reported in mg of gallic acid per g of n/m BP.

The efficacy of n/m BP in scavenging DPPH (2,2 diphenyl‐1‐picryl‐hydrazyl) radicals was determined as described by Brand‐Williams et al. (1995); Akowuah et al. (2005); de Florio Almeida et al. (2017). First, 0.5 mL of different dilutions of n/m BP (0.15, 0.31, 0.62, 1.25, 2.5, 5, 10, 20, and 40 mg/mL) were mixed with 0.3 mL of 0.5 mM DPPH solution in methanol and 3.0 mL of methanol, and then incubated at 23°C in the dark for 45 min. The absorbance of the solutions was determined using a UV–VIS spectrophotometer (Model XD7500, LOVIBOND, Dortmund, Germany) at 517 nm. The EC_50_ (mg/mL) is the n/m BP concentration that scavenges 50% of the free DPPH radicals, according to using Equation (1).

(1) $$\text{Inhibition percent}=\frac{\mathrm{AC}-\mathrm{AS}}{\mathrm{AC}}\times 100$$

AC = absorbance of the control sample; AS = absorbance of the test sample.

### 2.5 Antibacterial activity assay of n/m BP

The antimicrobial activity of n/m BP, minimum inhibitory concentration (MIC_n/m BP_) and minimum bactericidal concentration (MBC_n/m BP_), was determined against *Bacillus cereus* PTCC 1247, *Staphylococcus aureus* PTCC 1189, *Pseudomonas aeruginosa* PTCC 1555, and *Escherichia coli* PTCC 1769 (Chen et al., 2019; Dorman & Deans, 2000; Herreros et al., 2005; Moradi et al., 2023). The pathogenic strains were supplied by the Iranian Research Organization for Science and Technology (IROST, Iran). All strains were grown in Mueller–Hinton broth (MHB) at 37°C for 18 h. For broth microdilution assay, n/m BP was dissolved in 99% DMSO and then serial dilution (final concentration of 62.5–8000 [μg/mL]) was performed using a 96‐well microplate. 100 μL of MHB and 100 μL of serial dilution of n/m BP were added to each well of the microplate. Bacterial inoculation was performed at a ratio of 1% (v/v) using overnight cultures of the indicator bacteria (adjusted to a 0.5 McFarland standard, 1.5 × 10^8^ CFU/mL). The microtiter plates with a final bacterial concentration of 10^6^ CFU/mL were then incubated at 37°C for 24 h. The MIC_n/m BP_ is the lowest concentration of n/m BP at which the bacteria in MHB cannot visibly grow. The well in which no bacterial growth was detectable was inoculated onto Mueller–Hinton agar (MHA). The MBC_n/m BP_ was defined as the lowest concentration of n/m BP that suppresses the ability of bacteria to form colonies after 24 h of incubation at 37°C.

### 2.6 Preparation of the high‐fat sausage

All sausage samples were prepared at the Mahisa meat products factory (Hamedan, Iran). Based on the results of microbiological control (MBC) of n/m BP and considering the negative effects of high concentrations of n/m BP on sausage flavor, it is suggested to use 125 and 250 mg of n/m BP as a functional ingredient with antioxidant and antimicrobial activities per 100 g of sausage. The percentage of ingredients in the basic formula for the sausages is as follows:

Lamb meat 38.6%, sheep's tail 9.68%, ice 27.02%, salt 1.06%, gluten 3.67%, wheat flour 5.45%, starch 7.24%, sodium phosphate 0.55%, carrageenan 0.27%, spices 1.64%, bell peppers 2.41%, garlic 2.41%, and sodium nitrite 100 ppm.

To evaluate the antioxidant effect of n/m BP in preventing lipid oxidation in high‐fat sausages, the sausage formulation was grouped into four parts. The first group, which served as a control, contained no antioxidant substance. The second, third, and fourth groups contained 125 mg n/m BP, 250 mg n/m BP, and 100 mg ascorbic acid per 100 g of meat, respectively. The sausage samples were cooked at 90°C for 15 min and then cooled to 25°C. The sausages were packed separately in plastic packaging (Arta food casings, Tabriz, Iran) and stored at 4°C for 30 days for further analysis. The sausage was aseptically sampled at three time intervals (10, 20, and 30 days) during storage for further analysis.

### 2.7 Physiochemical properties of the sausages

The sausage samples were analyzed during the storage period using standard methods (ISIRI, 2020a, 2020b) for physicochemical properties, including dry weight percentage (DW%) and pH. The DW% of the sausages was determined by moisture loss after drying at 103°C in an oven (Parsian Teb, Tehran, Iran) to a constant mass during the storage period. The pH values of the sausages were measured using a pH meter (827 pH Lab, Metrohm, Herisau, Switzerland). First, the pH meter was calibrated with a buffer for pH values of 4 and 7. A quantity of 10 g of samples was mixed with distilled water (100 mL) until a homogeneous solution was obtained, and then the pH of the samples was measured at room temperature (about 23°C).

### 2.8 Oxidative stability of sausages

The test for thiobarbituric acid reactive substances (TBARS) was carried out as previously described by Raharjo et al. (1992); Botsoglou et al. (1994); Ulu (2004); Alirezalu et al. (2017); de Florio Almeida et al. (2017) with some modifications. For the extraction of the aldehydes, 2 g of the sausage was mixed with 8 mL of 5% trichloroacetic acid and 5 mL of a 0.004% BHT solution in hexane. This mixture was homogenized for 30 s and then centrifuged at 4000 *g* for 5 min. After centrifugation, the aqueous intermediate solution containing malondialdehyde was removed and filtered. 2 mL of the filtered solution was reacted with 1.5 mL of TBARS (0.02 mol/L) and 0.5 mL of 5% trichloroacetic acid at 85°C for 40 min. The absorbance of the samples was determined in comparison to the control using a UV–VIS spectrophotometer (model XD7500, LOVIBOND, Dortmund, Germany) at 532 nm. The control sample contained distilled water (5 mL) and 5% aqueous TBARS solution (5 mL). The results of TBARS values were reported as mg malonaldehyde (MDA)/kg sample. TBARS were measured using a standard curve of 5 to 40 nmol/L malondialdehyde (Tran et al., 2020).

### 2.9 Microbiological analysis

After homogenization of the sausage with a bag mixer/stomacher (BagMixer Lab Blender, 400 W; Interscience, Saint‐Nom‐la‐Bretèche, France) for 2 min under sterile conditions, a serial dilution was performed to a dilution of 10^−7^ with sterile peptone water. The corresponding dilutions of the samples were plated on Violet Red Bile Lactose Agar (VRBA) for coliform bacteria, on Plate Count Agar (PCA) for the total bacterial count, on Baird Parker Agar (BPA) for *S. aureus* and on Potato Dextrose Agar (PDA) for fungi. PCA and BPA plates were incubated at 30°C for 48 h, VRBA and PDA were incubated at 37°C for 48 h and at 25°C for 3–5 days, respectively (Downes & Ito, 2001; ISIRI, 2005, 2015; Pommerville, 2007).

### 2.10 Sensory analysis

The sensory properties of the sausage samples were analyzed at the end of the 30‐day storage period. The samples were evaluated for taste, texture, color, and overall acceptability on the 5‐point hedonic scale. For the sensory analysis, 40 subjects (20 women and 20 men) aged 18–30 years were selected and trained. The participants evaluated the sensory properties of the sausage samples at room temperature (20–25°C).

### 2.11 Statistical analysis

Statistical analysis was carried out using two‐way ANOVA (SPSS version 16 software) followed by LSD test for statistical significance of *p* < .05. The effects of formulation with BP (0, 125 and 250 mg/100 g meat) and ascorbic acid (100 mg/100 g meat) during the storage period (0, 10, 20 and 30 days) on %DW, TBA, pH, microbial count and sensory properties were evaluated. Results were expressed as mean ± SD and all experiments were performed in triplicate.

## 3 RESULTS AND DISCUSSION

### 3.1 Morphology of n/m BP

**FIGURE 1:**
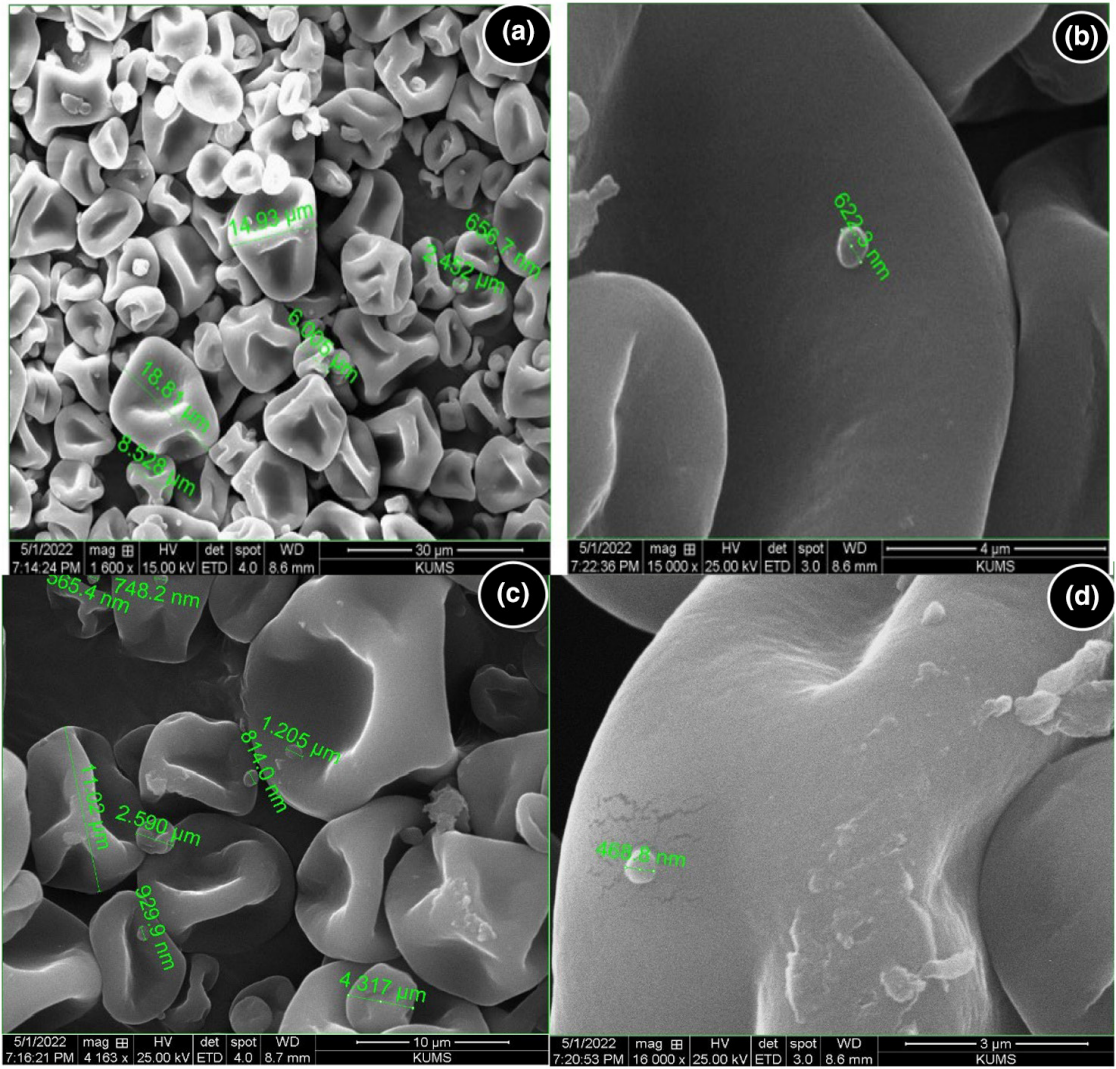
SEM images of n/m BP prepared at 1600× magnification (a), 15,000 × magnification (b), 4163 × magnification (c), and 16,000 × magnification (d).

The formation of BP particles produced by spray drying at the nano/microscale was confirmed by SEM (Figure 1). The formation of microscale BP particles (1.205–18.81 μm) contained several voids and nanoscale BP particles (468.8–929.9 nm) in spherical shape was remarkable. Since the formation of nanoparticles on the nanoscale is better below 100 nm, it makes sense to consider BP particles on the nano/microscale in the present study. In general, BP occurs in the form of grains with a size of 2.5–250 μm (Komosinska‐Vassev et al., 2015). However, drying methods such as freeze‐drying and spray‐drying can also affect the particle size of bee products. In previous studies, the average size of propolis microparticles was measured at 4.06 μm (Dota et al., 2011), 10–90 μm (Pant et al., 2022), and 50–5000 μm (Mangiring et al., 2018) produced by spray drying, vacuum drying, and freeze drying, respectively.

### 3.2 TPC and antioxidant activity of n/m BP

Phenolic compounds as secondary metabolites of plants are classified into two groups based on the number of phenolic units in their molecules: simple phenols and polyphenols, which have antioxidant properties due to their redox activity. The TPC of n/m BP was 28.26 ± 0.10 mg GAL/g pollen. Our results showed that n/m BP had a higher TPC than the results of de Florio Almeida et al. (2017) (19.69 ± 0.14 mg GAE/g); Jalili et al. (2022) (2.79 to 11.83 mg/ GAE/g) and Novaković et al. (2021) (13.04 ± 1.31 mg GAE/g). However, Leja et al. (2007); Ulusoy and Kolayli (2014); Pascoal et al. (2014); Anjos et al. (2019) reported high levels of TPC for BP.

The result of the antioxidant activity of n/m BP by DPPH assay was expressed in terms of EC_50_ value. The EC_50_ value of n/m BP was 5.4 ± 0.07 mg/mL. The antioxidant activity of n/m BP may be associated with the presence of phenolic compounds in BP. In previous studies, the EC_50_ value (mg/mL) of BP extracts from different locations of the world was reported as 0.97 mg/mL (de Florio Almeida et al., 2017), 2.62 ± 0.09 mg/mL (Anjos et al., 2019), 0.18 mg/mL (Graikou et al., 2011), and 2.16 to 5.87 mg/mL (Morais et al., 2011). The origin of the BP, the type of analytical method used to determine the antioxidant activity, and the geographical location of the BP collection are effective factors for this comparison (de Florio Almeida et al., 2017). Therefore, the results of the antioxidant activity of BP are expected to vary in different studies. BP contains a broad spectrum of phenolic compounds that have antioxidant activity and can chelate metal ions. The antioxidant property of phenolic compounds is attributed to their oxidizing and reducing activities. LeBlanc et al. (2009) pointed out that the strong antioxidant activity of BP in inhibiting DPPH‐free radicals is due to the polyphenolic flavonoids (quercetin and naringenin). There is also a correlation between the antioxidant potential of BP and the other phenolic compounds such as trans‐cinnamic acid, vanillic acid, p‐coumaric acid, caffeic acid, ferulic acid, cis, trans‐abscisic acid, rutin, syringic acid, and *p*‐OH benzoic acid. By increasing the phenolic compounds in BP, the antioxidant activity and the inhibition of free radicals increase (Kroyer & Hegedus, 2001; Ulusoy & Kolayli, 2014).

### 3.3 Antibacterial activity of n/m BP

The antimicrobial activity of n/m BP against food‐borne bacteria was confirmed according to Table 1. In similar studies, BP has shown antibacterial activity against *S. aureus*, *B. cereus*, *E. coli*, and *P. aeruginosa* (Abouda et al., 2011; Bridi et al., 2019; Cabrera & Montenegro, 2013; Didaras et al., 2020; Graikou et al., 2011; Khider et al., 2013; Morais et al., 2011; Pascoal et al., 2014). As shown in Table 1, the MIC_n/m BP_ for all test bacteria was 1000 μg/mL. The results showed that Gram‐positive bacteria were more sensitive compared to Gram‐negative bacteria, such that *P. aeruginosa* (MBC_n/m BP_: 4000 μg/mL) and *E. coli* (MBC_n/m BP_: 4000 μg/mL) were the most resistant bacteria, while *B. cereus* (MBC_n/m BP_: 2000 μg/mL) and *S. aureus* (MBC_n/m BP_: 2000 μg/mL) were the most sensitive bacteria. According to Morais et al. (2011); Abouda et al. (2011); Graikou et al. (2011); Pascoal et al. (2014); Karadal et al. (2018); Urcan et al. (2018); Didaras et al. (2020); Ilie et al. (2022), bee products are more effective against Gram‐positive bacteria than against Gram‐negative bacteria, with a few exceptions reported by Fatrcová‐Šramková et al. (2013); AbdElsalam et al. (2018); Šimunović et al. (2019). The antimicrobial activity of BP can be attributed to the chemical compounds and their synergistic effects. Therefore, it is difficult to precisely attribute the antibacterial activity of BP to a specific compound or group of compounds, as BP has a complex matrix with different chemical compositions (Ilie et al., 2022). Ilie et al. (2022) reported that *P. aeruginosa* was resistant to chlorogenic acid, apigenin kaempferol, and quercetin in BP. In general, it is known that Gram‐negative bacteria are less sensitive to the inhibitory effect of most antimicrobial compounds because, in addition to the peptidoglycan–lipoprotein complex in their cell wall, they also have an outer membrane that acts as a strong barrier to permeability (Morais et al., 2011; Santa Bárbara et al., 2021).

**TABLE 1:** MIC and MBC of n/m BP against food‐borne bacteria.

Microorganisms	MIC (μg/mL)	MBC (μg/mL)
*Staphylococcus aureus* ATCC 29213	1000	2000
*Bacillus cereus* ATCC 11778	1000	2000
*Pseudomonas aeruginosa* ATCC 9027	1000	4000
*Escherichia coli* ATCC 25922	1000	4000

In general, the antimicrobial activity of BP can be attributed to the presence of its antimicrobial compounds such as polyphenols (Cushnie & Lamb, 2011; Daglia, 2012), fatty acids (Yoon et al., 2018), alkaloids (Lee et al., 2016), and flavonoids (Didaras et al., 2020). Previous studies have confirmed the antimicrobial activities of the flavonoid compounds found in BP as follows:

Glycosides damage the membrane and cell wall of bacteria, interfere with the transport process and motility of bacteria (Wang et al., 2018), control the biofilm formation of fungi (Rocha et al., 2019), and inhibit the activity of topoisomerase IV (Liu, Otsuka, et al., 2009); kaempferol controls the biofilm formation of fungi (Rocha et al., 2019); myricetin inhibits the activity of DnaB helicase in *E. coli* (Griep et al., 2007); luteolin impairs the activities of cell membranes and acts as an anti‐biofilm in bacteria (Qian et al., 2020); apigenin destabilizes the components of the cell wall in bacteria (Hariri et al., 2017; Wu et al., 2008); galangin leads to aggregation of the cells bacteria (Cushnie et al., 2007), damage to the cytoplasmic membrane and loss of potassium (Cushnie & Lamb, 2005). In addition, the antimicrobial activity of phenolic compounds found in BP has been demonstrated as follows: Ferulic acid and gallic acid cause rupture of the cell membranes of bacteria and alter their surface hydrophobicity (Borges et al., 2013); caffeic acid esters prevent bacterial growth through an oxidative stress process (Collins et al., 2019); p‐coumaric acid damages the cell membranes of bacteria and disrupts their genome structure by binding to DNA (Lou et al., 2012).

### 3.4 Physiochemical properties of the sausages

**FIGURE 2:**
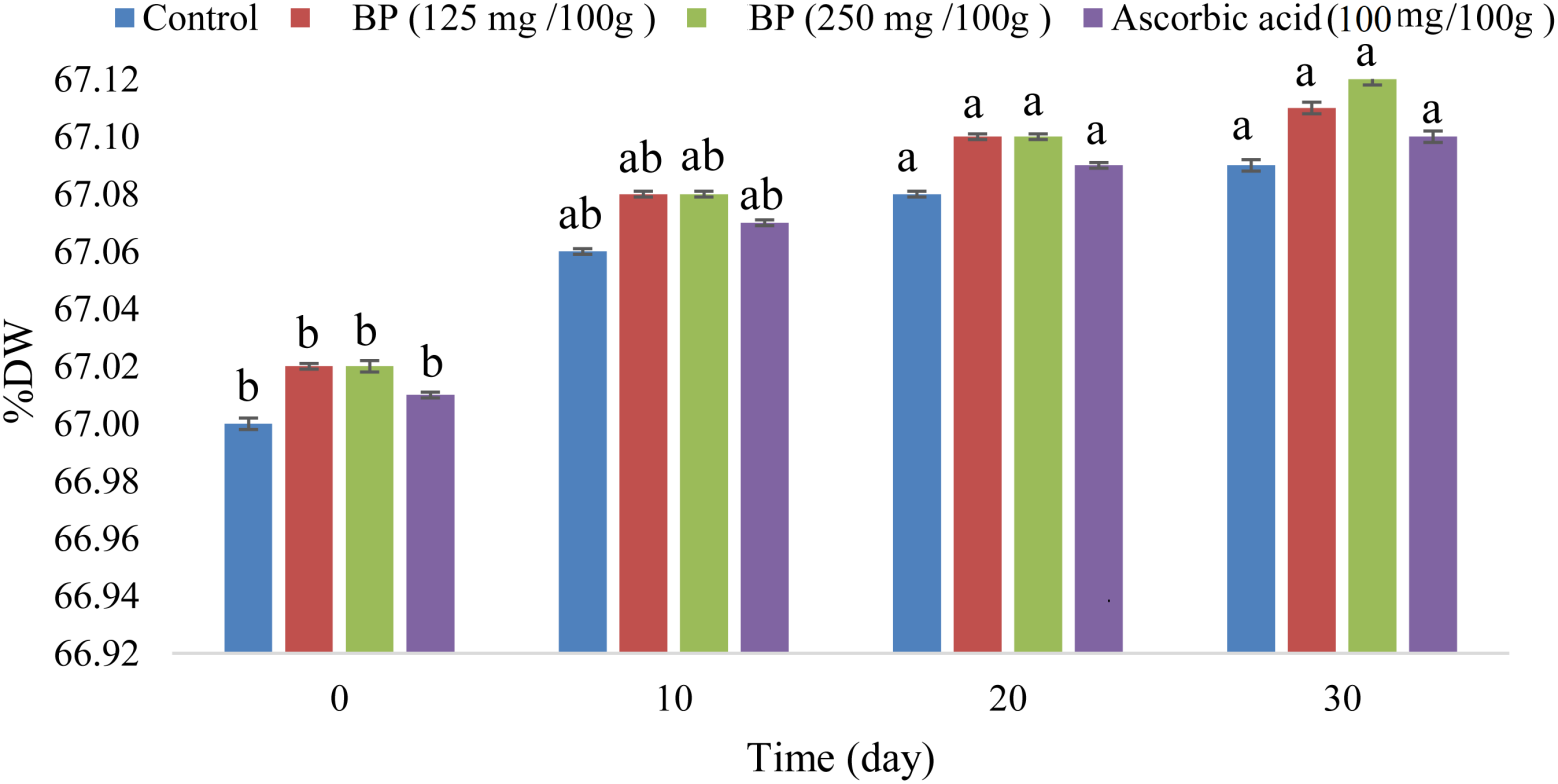
Effect of n/m BP on the DW% in sausage samples. Vertical bars represent the standard deviation (*n* = 3). Different letters indicate statistically significant differences (*p* < .05).

As can be seen in Figure 2, there was no significant difference (*p* < .05) in DW% between the different sausage formulations during the storage period. The results showed that the addition of n/m BP slightly increased the DW% of the sausage samples compared to the other samples. The DW% increased slightly in all samples during the storage period. The highest DW% (67.12%) was obtained for the samples with n/m BP (250 mg/100 g meat) on the 30th day of the storage period, while the lowest DW% (67%) was obtained for the control sample on the 0th day of the storage period. This finding is in line with the results of Radulović et al. (2011); Li et al. (2013); Zhang et al. (2017); Novaković et al. (2021).

**FIGURE 3:**
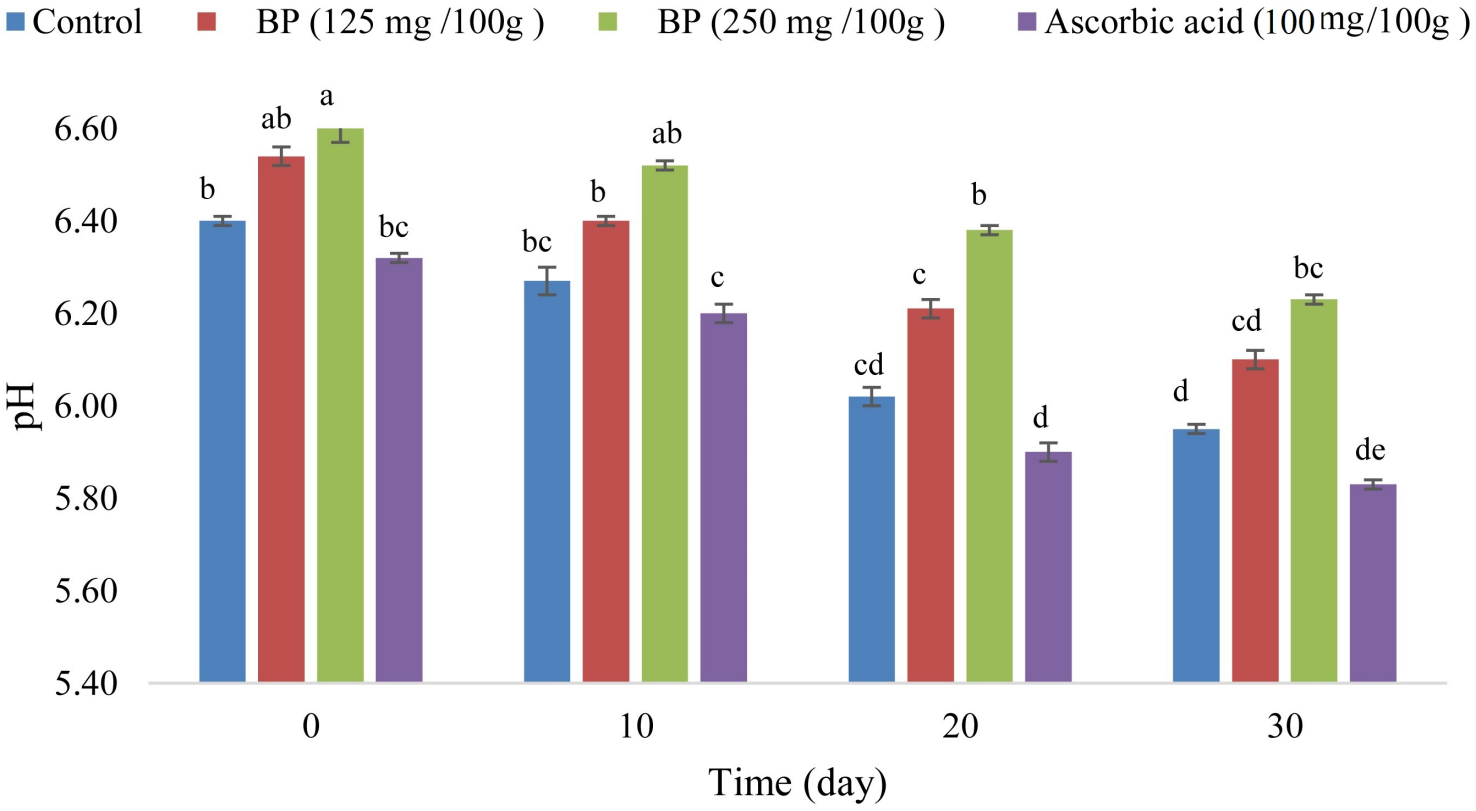
Effect of n/m BP on the pH in sausage samples. Vertical bars represent the standard deviation (*n* = 3). Different letters indicate statistically significant differences (*p* < .05).

The pH values of the sausage samples were in the range between 5.83 and 6.60, values that correspond to the ISIRI (2021). As can be seen in Figure 3, there was a significant difference (*p* < .05) in pH values between the different sausage formulations and the addition of n/m BP resulted in a significant increase in pH values. The pH value of the different formulations of the sausage samples was influenced by the added antioxidant and the storage time. The pH reduction during storage period can be attributed to the fermentation of carbohydrates and subsequent lactic acid production by lactic acid bacteria (LAB) (Wójciak et al., 2014). These results are consistent with previous studies by Bozkurt (2006); Comi et al. (2015); Anjos et al. (2019); Novaković et al. (2021). The sausage samples with n/m BP (250 mg/100 g meat) as a natural antioxidant had the highest pH (6.60) on day 0 of storage and the sausage samples with ascorbic acid (100 mg/100 g meat) as an industrial antioxidant had the lowest pH (5.83) on day 30 of storage. These variations could be due to the low pH of ascorbic acid leading to a decrease in pH in the sausage sample compared to the other samples. In addition, the current study showed that n/m BP has antimicrobial activity. Therefore, it was expected that by increasing the percentage of n/m BP in the sausage samples, the microbial growth and subsequently the production of organic acids would decrease, thereby increasing the pH. Similarly, previous studies have also confirmed the antimicrobial activity of BP extract (Didaras et al., 2020; Fatrcová‐Šramková et al., 2013; Kaškonienė et al., 2020; Zlatev et al., 2018).

### 3.5 TBARS

**FIGURE 4:**
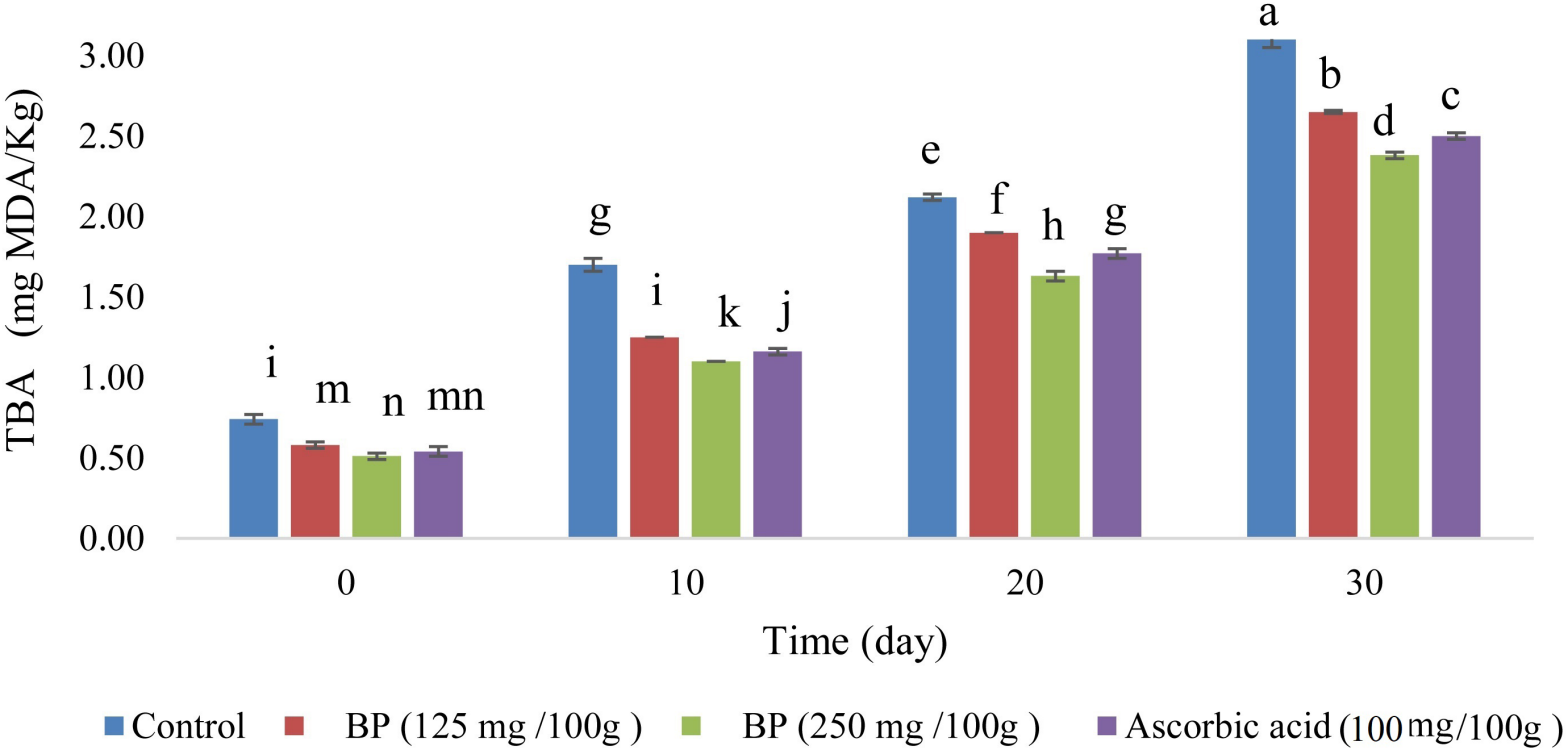
Effect of n/m BP on TBARS in sausage samples. Vertical bars represent the standard deviation (*n* = 3). Different letters indicate statistically significant differences (*p* < .05).

TBARS is an indicator of the progression of lipid peroxidation in meat products and expresses the level of MDA generated by the oxidation of UFA (Tang et al., 2001). As can be seen in Figure 4, there was a significant difference (*p* < .05) in TBARS between the different sausage formulations, and this indicator increased significantly during the storage period. In addition, the results showed that the addition of n/m BP decreased the value of MDA/kg sausage compared to the control sample. The lowest value for malonaldehyde (0.51 mg MDA/kg sample) was found for the samples with n/m BP (250 mg/100 g meat) on day 0 of the storage period, while the highest value (3.10 mg MDA/kg sample) was found for the control sample on day 30. As a result, the sample with n/m BP (250 mg/100 g meat) had lower TBARS levels than the other sausages during each storage period. This finding is consistent with the results of de Florio Almeida et al. (2017); Alirezalu et al. (2019); Anjos et al. (2019); Novaković et al. (2021). It should be noted that the malonaldehyde content was different in meat products treated with BP antioxidants, which may be due to the different spices, lipid composition, and muscle type in the meat products (Elimam & Mohammed, 2013). Selani et al. (2011) reported that a meat product with an MDA content of less than 3 mg/kg was produced and stored under good conditions. According to current results, the sausage samples with n/m BP would be considered to be in good condition during storage (2.38–2.65 mg MDA/kg sample on the 30th day). BP contains carotenoids and phenolic compounds that can be gradually transferred from the pollen grain to the meat product and act as free radical scavengers to prevent further lipid oxidation (Novaković et al., 2021). The results showed that the n/m BP antioxidants react with the free radicals in the high‐fat sausage, limiting the progression of lipid oxidation during the storage period. The current results show that the content of TPC (28.26 ± 0.10 mg GAL/g) was considerable as a source of antioxidants in n/m BP. Moreover, the strong antioxidant property of n/m BP might be related to the small size, large solubility, and good permeability of the nano‐ and microparticles of BP; consequently, free radicals are effectively involved in the scavenging effect of BP particles (Lou et al., 2012; Moradi et al., 2023). Since n/m BP gradually releases bioactive chemicals, it was expected to have a strong antioxidant effect in high‐fat sausage, especially in sausages with n/m BP (250 mg/100 g meat). Furthermore, the results confirmed that the antioxidant activity of n/m BP (250 mg/100 g meat) is more effective than that of ascorbic acid (a commercial antioxidant). Similarly, several studies have confirmed the effective role of natural antioxidants in preventing lipid peroxidation in meat products (Alvarez‐Parrilla et al., 2014; Kim et al., 2013; Lara et al., 2011; Sampaio et al., 2012). Among natural antioxidants, plant products have a significant effect on the control of lipid oxidation of meat products, such as resveratrol and thymol in cooked sausage (Hashemi et al., 2023), resveratrol and essential oil of *Satureja bachtiarica* in fresh chicken meat (Abdalbeygi et al., 2022), essential oil of *Ziziphora tenuior* and orange fiber in beef cooked sausage (Aminzare, Hashemi, Afshari, Mokhtari, & Noori, 2022).

### 3.6 Microbiological analysis

**FIGURE 5:**
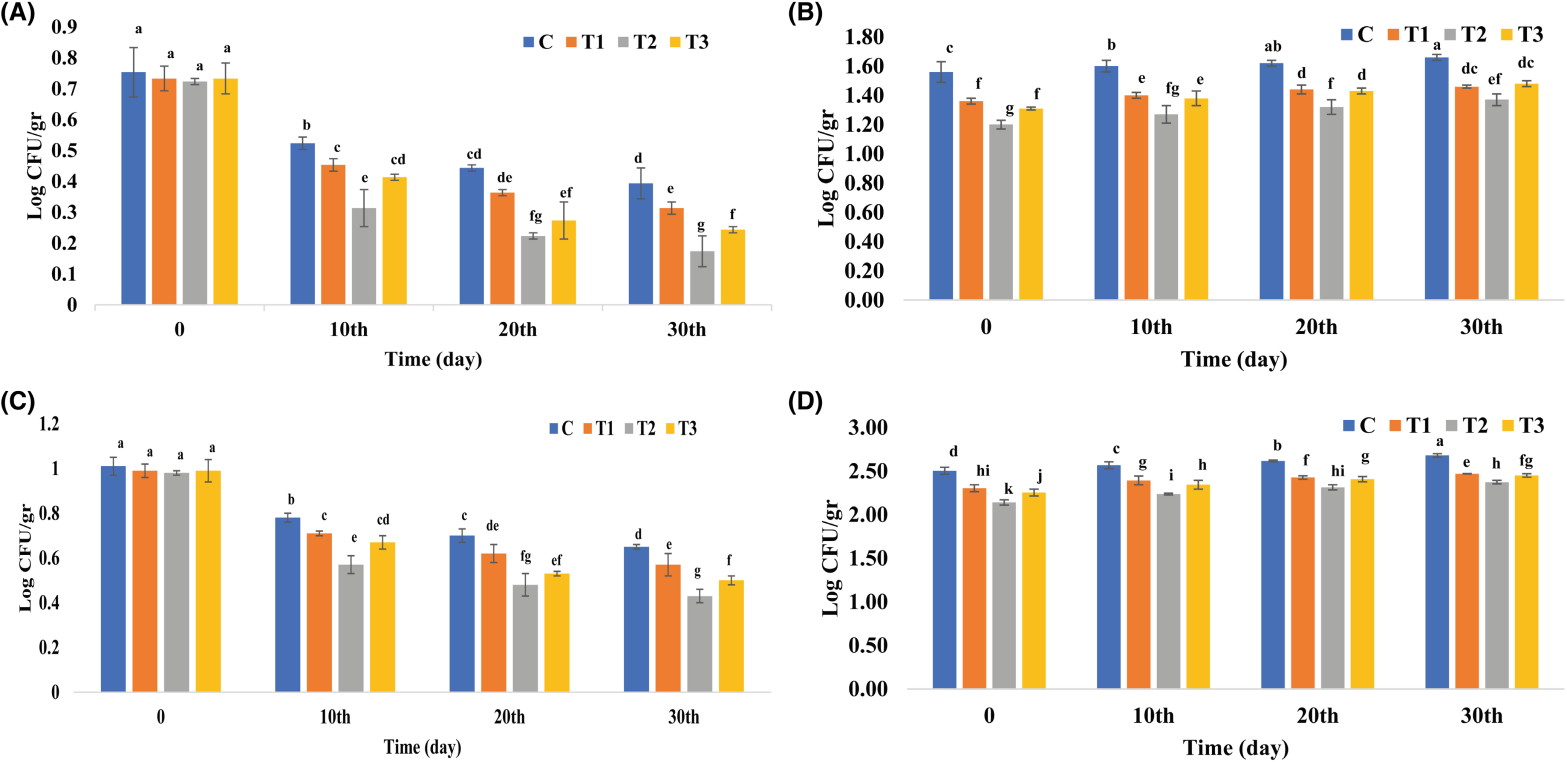
Effect of n/m BP on the microbial growth in sausage samples during 30 days of storage. Vertical bars represent the standard deviation (*n* = 3). Different letters indicate statistically significant differences (*p* < .05); (A) *S. aureus*; (B) Coliform; (C) Fungi; (D) total bacterial count. C, control; T1, Sausage with n/m BP (125 mg/100 g meat); T2, Sausage with n/m BP (250 mg/100 g meat); T3, Sausage with ascorbic acid (100 mg/100 g meat).

As can be seen in Figure 5, the microbial growth in the sausage samples changed during the 30‐day storage period. During the storage period, the coliforms and the total bacterial count increased, while the number of fungi and *S. aureus* decreased in all samples. The greater reduction in the number of *S. aureus* and fungi in high‐fat sausages with n/m BP compared to the control sample during storage can be attributed to the strong antimicrobial and antifungal effect of BP against Gram‐positive bacteria and the fungal community, respectively (Almaraz‐Abarca et al., 2004; Komosinska‐Vassev et al., 2015). The coliform bacteria and the total bacterial count tended to increase during the 30‐day storage period in sausage samples. This trend could be due to the ingredients added to the meat products (Fernández‐López et al., 2019). In general, microbial growth was influenced by the antimicrobial activity of n/m BP, as the number of microorganisms in the samples decreased with increasing n/m BP concentration. In addition to the results of the current study, numerous scientific reports have confirmed that BP has considerable antimicrobial activity against microorganisms (Graikou et al., 2011; Pascoal et al., 2014). As mentioned earlier, the antimicrobial activity of BP can be attributed to the presence of its antimicrobial ingredients, including polyphenols, fatty acids, alkaloids, and flavonoids. Graikou et al. (2011) showed that the antibacterial property of BP is due to the high concentration of quercetin. In addition, the control of microbial growth may be related to the easy distribution of the lipophilic molecules of BP in the fat‐rich matrix, which is common in meat products (Novaković et al., 2021). It should be noted that the significant antimicrobial effect of n/m BP may also be related to the reduction in the size of pollen particles at the nano‐ and micro‐scale, making the antimicrobial activity of BP particles more effective due to the larger contact area. This finding is in line with de Souza Ferreira et al. (2014); Jansen‐Alves et al. (2019); Shubharani et al. (2019); Jin and Jin (2021); Moradi et al. (2023). In addition to BP, the antimicrobial effect of other natural preservatives to control microbial growth in meat products has also been studied several times. Hashemi et al. (2023) showed that a film based on sodium alginate with resveratrol and thymol can control the growth of *L. monocytogenes* in cooked sausages. Aminzare, Hashemi, Afshari, Noori, and Rezaeigolestani (2022) increased the shelf life of cooked sausage with dietary fibers. In addition, Abdalbeygi et al. (2022) controlled the growth of *E. coli* in fresh chicken with chitosan, resveratrol and essential oil of *Satureja bachtiarica*; Abbasi et al. (2021) controlled the growth of *L. monocytogenes* in fresh chicken meat using a nano emulsion of *Zataria multiflora* essential oil together with cinnamaldehyde; Aminzare, Hashemi, Afshari, Mokhtari, and Noori (2022) showed that microencapsulated essential oil of *Ziziphora tenuior* and orange fiber were effective in controlling the sanitary quality of cooked beef sausage. Consequently, the addition of n/m BP can be effective in controlling microbial growth in meat products and the current results are in line with the results of Turhan et al. (2014); Turhan et al. (2017); Anjos et al. (2019); Kostić et al. (2020); Novaković et al. (2021).

### 3.7 Sensory analysis of sausage samples

One of the limitations of using plant extracts and essential oils in food is their influence on the sensory attributes of the product (Moradi et al., 2023). BP may influence the sensory properties of food due to its special color and taste. In the current study, the effect of different levels of n/m BP on the color, taste, texture, and overall acceptance of sausage samples after 30 days was investigated. This corresponds to the legal shelf life for industrial production.

**FIGURE 6:**
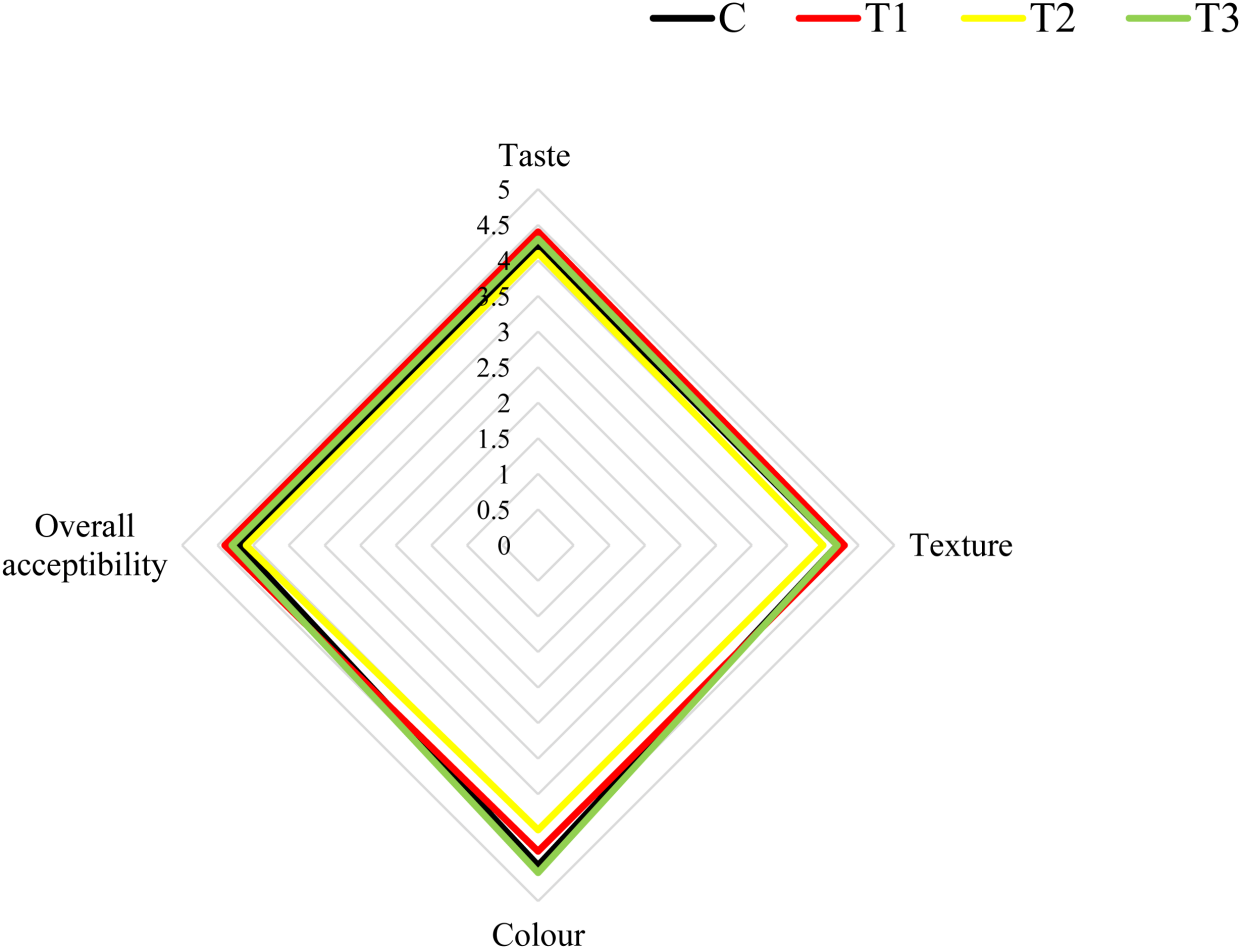
Consumer acceptability scores on a 5‐point scale for sausage samples in the third day. C, control; T1, Sausage with n/m BP (125 mg/100 g meat); T2, Sausage with n/m BP (250 mg/100 g meat); T3, Sausage with ascorbic acid (100 mg/100 g meat).

As shown in Figure 6 (Table 2), T1 (sample with n/m BP 125 mg/100 g meat) received the highest rating for texture (4.3), taste (4.4), and overall acceptability (4.4), and the lowest score for texture (4), taste (4.1), color (4), and overall acceptability (4.1) was obtained for T2 (sample with n/m BP 250 mg/100 g meat), after 30 days of storage. The results showed that the sensory properties of the sausage samples were affected by n/m BP and that higher concentrations resulted in unpleasant taste and undesirable color.

**TABLE 2:** Effect of n/m BP on the sensory properties of the sausage samples after 30 days of storage.

Formulation	Taste	Texture	Color	Overall acceptability
C	4.2 ± 0.52^bc^	4.2 ± 0.32^bc^	4.5 ± 0.49^b^	4.2 ± 0.47^b^
T1	4.4 ± 0.51^a^	4.3 ± 0.38^a^	4.3 ± 0.49^c^	4.4 ± 0.52^a^
T2	4.1 ± 0.49^c^	4 ± 0.43^c^	4 ± 0.45^d^	4.1 ± 0.49^c^
T3	4.3 ± 0.49^ab^	4.2 ± 0.43^ab^	4.6 ± 0.51^a^	4.3 ± 0.49^ab^

*Note*: Different letters in each column (*n* = 3) indicate statistically significant differences (*p* < .05).

Abbreviations: C, control; T1, Sausage with n/m BP (125 mg/100 g meat); T2, Sausage with n/m BP (250 mg/100 g meat); T3, Sausage with ascorbic acid (100 mg/100 g meat).

After 30 days of storage, the values of the sensory attributes taste, texture, and overall acceptability were low for the control sample compared to T1 (sample with n/m BP 125 mg/100 g meat). The presence of hydroperoxides leads to unpleasant odor and rancidity in high‐fat foods (Alizadeh et al., 2019). Thus, the decreasing trend in the ratings of taste and overall acceptability in the control sample could be related to lipid oxidation (rancidity) during the 30‐day storage; however, in the samples with n/m BP, these oxidative reactions were inhibited by the antioxidant activity of n/m BP. Similarly, Novaković et al. (2021) showed that the panelists detected no change in odor and taste due to lipid oxidation in BP‐containing frankfurters, while there was a significant difference in the color of the samples. However, Anjos et al. (2019) reported that the addition of BP to black pudding as a meat product did not affect the sensory properties of the samples. From these results, it can be concluded that the low level of n/m BP effectively improves the taste, texture, and overall acceptability of the sausage compared to other samples.

## 4 CONCLUSION

Finding a powerful natural antioxidant as a suitable alternative to synthetic antioxidants has always been a challenge for the food industry, especially for meat products. This study shows that the use of nano/microparticles of BP as a functional natural ingredient with antioxidant and antimicrobial activities can be an effective approach to maintain the quality of high‐fat sausage during storage. It should be noted that the presence of lipophilic compounds in BP should not be neglected, as these compounds are easily distributed in the fat matrix of high‐fat meat products and exert an effective antioxidant action to delay lipid oxidation during storage time. In this study, the remarkable properties of nano/microparticles of BP in controlling lipid peroxidation and microbial growth of sausage can be attributed to the presence of potential antioxidant compounds, better availability of bioactive compounds with antimicrobial and antioxidant activities due to smaller size and easy solubility in the fat matrix. Therefore, the addition of n/m BP is recommended as a suitable alternative to synthetic antioxidants to improve the quality of high‐fat sausage and extend its shelf life. Since most studies have investigated the effect of natural size BP on the retardation of lipid oxidation in meat products, this study only investigated the effect of nano/microparticle size BP particles on the oxidative stability of high‐fat sausage. Therefore, it is suggested that future studies compare the effects of natural size and smaller size BP particles (nano‐ and microparticles) on the oxidative stability of high‐fat sausages.

## AUTHOR CONTRIBUTIONS

**Zahra Mashhadi:** Conceptualization (equal); data curation (equal); formal analysis (equal); investigation (equal); methodology (equal); software (equal). **Nafiseh Davati:** Conceptualization (lead); data curation (lead); formal analysis (lead); funding acquisition (supporting); investigation (lead); methodology (lead); project administration (lead); resources (supporting); software (lead); supervision (lead); validation (lead); visualization (lead); writing – original draft (equal); writing – review and editing (equal). **Aryou Emamifar:** Methodology (lead); project administration (supporting); supervision (lead). **Mostafa Karami:** Methodology (lead); project administration (supporting); supervision (lead).

## FUNDING INFORMATION

The authors did not receive support from any organization for the submitted work.

## CONFLICT OF INTEREST STATEMENT

The authors declare that they do not have any conflict of interest.

## ETHICAL STATEMENT

This study does not involve any human or animal testing.

## CONSENT TO PARTICIPATE

All the coauthors are willing to participate in this manuscript.

## CONSENT FOR PUBLICATION

All authors are willing to publish this manuscript and they are willing to contribute to this manuscript.

## Data Availability

Even though adequate data have been given in the form of tables and figures, all authors declare that if more data are required, then the data will be provided on a request basis.
